# On the use of the P300 as a tool for cognitive processing assessment in healthy aging: A review

**DOI:** 10.1590/1980-57642018dn12-010001

**Published:** 2018

**Authors:** Sofia Cristina Iost Pavarini, Allan Gustavo Brigola, Bruna Moretti Luchesi, Érica Nestor Souza, Estefani Serafim Rossetti, Francisco José Fraga, Letícia Pimenta Costa Guarisco, Marélli Terassi, Nathalia Alves Oliveira, Priscilla Hortense, Renata Valle Pedroso, Ana Carolina Ottaviani

**Affiliations:** 1 MS, Federal University of São Carlos (UFSCar), Graduate Program in Nursing, São Carlos, SP, Brazil.; 2 PhD, Federal University of São Carlos (UFSCar), Graduate Program in Gerontology, São Carlos, SP, Brazil.; 3 PhD, Federal University of ABC (UFABC), Engineering, Modeling and Applied Social Sciences Center (CECS), Santo André, SP, Brazil.; 4 PhD, Federal University of São Carlos (UFSCar), Gerontology Department, São Carlos, SP, Brazil.; 5 PhD, São Paulo State University (UNESP), Physical Activity and Aging Lab, Rio Claro, SP, Brazil.

**Keywords:** aging, neuropsychological function, event-related potentials, P300, healthy elderly, envelhecimento, função neuropsicológica, potencial relacionado a eventos, P300, idosos saudáveis

## Abstract

Changes in patterns of performance for the cognitive functions of memory, processing speed, and focused attention are expected in old age.

**Objective:**

The main goal of this systematic review was to analyze the use of ERP in healthy elderly in studies evaluating the P300 components.

**Methods:**

A systematic review was carried out based on recommendations for nursing research on the databases LILACS, PsycINFO, PubMed, SCOPUS and *Web of Science*.

**Results:**

26 studies involving 940 healthy elderly were identified, most of which sought to identify and determine the influence of age on the P300.

**Conclusion:**

Although there is consensus in the literature that P300 latency is significantly longer in elderly with psychiatric disorders compared to healthy elderly, it was not possible to conclude P300 associations with gender, education and other cognitive tests.

## RESUMO

São esperadas mudanças nos padrões de desempenho nas funções cognitivas de memória, velocidade de processamento e atenção concentrada durante o processo de envelhecimento.

**Objetivo:**

O objetivo principal desta revisão sistemática é analisar o uso de ERP em idosos saudáveis nos estudos que avaliaram os componentes do P300.

**Métodos:**

Realizou-se uma revisão sistemática com recomendações de estudo para enfermagem nos bancos de dados LILACS, PsycINFO, PubMed, SCOPUS and *Web of Science*.

**Resultados:**

Foram identificados 26 estudos com 940 idosos saudáveis, a maioria procurava identificar e encontrar a influência da idade no P300.

**Conclusão:**

Embora haja consenso na literatura de que em idosos com transtornos psiquiátricos a latência P300 é significativamente maior, em relação aos idosos saudáveis não foi possível concluir as associações P300 com gênero, escolaridade e outros testes cognitivos.

Some age-related changes in brain function are expected. Therefore, changes in attention, concentration and memory may influence cognitive processing^1^ and because they are associated with these functions, endogenous event-related potentials (ERP) have been used to supplement neuropsychological evaluation. Endogenous ERP do not depend only on the individual's physical attributes, but also on brain reactions.^2^ The ERP are magnetic waves (measured by magnetoencephalography - MEG), where the third positive wave is known as P3 or P300, since it occurs around 300 milliseconds (ms) after the presentation of the stimulus. Two variables are used to quantify the P300: latency, which is related to information processing time, and amplitude, which is related to attention level.^3^ To evaluate the classic P300 (P3b), the oddball paradigm is usually used, by which subjects are instructed to discriminate the rare stimulus while irrelevant stimuli are also being presented. Tasks can be auditory, with stimuli at different sound frequencies, for instance, or visual, entailing presentation of figures.^4^^,^ 5

The variation in cognitive processing in the elderly, according to the literature, ranges from 300 ms to 400 ms or longer. There is evidence that a reduction in cognitive processing speed occurs with aging, characterized by increased latency and decreased amplitude.^2^^,^ 5 ^-^ 9

The literature indicates that cognitive processing can be influenced by factors such as age, gender, education, reading habits, cognitive performance, the presence of depressive symptoms, level of social and cultural interaction, among other aspects.^10^^,^ 11 A meta-analysis study found that P300 is a sensitive tool for monitoring cognition and may be an indicator in the analysis of cognitive deterioration.^4^

Given the above, the need to know and systematize the data on the use of the P300 for cognitive processing assessment in healthy elderly was recognized, considering that reviews published to date have focused on the association between age and cognitive processing in elderly with cognitive impairment.

The main goal of this systematic review was to analyze the use of ERP in healthy elderly in studies evaluating the P300 components, primarily the classical P300, with a focus on attention and cognitive processing speed. The intention was to identify the most relevant and recent output on the subject and provide information that can help guide the use of the P300 in elderly as a tool for cognitive processing assessment, both for clinical practice and research. Besides the above-mentioned objective, this study also aimed to identify evidence of the relationship between P300 and associated factors (age, gender, and education) in healthy aging.

## METHODS

This was a systematic review of the literature, carried out according to the stages recommended in the literature.^12^ The guiding questions that underpinned this review were "What are the characteristics about the recent output using the P300 for cognitive processing assessment in healthy elderly?" and "Can individual associated factors such as age, gender and education influence P300 in healthy elderly? What is the scientific evidence?".

The inclusion criteria were studies that used the P300 as a tool for evaluating cognitive processing in the elderly and that included at least one group of cognitively healthy elderly in the sample; studies with a publication date of between January 2011 and January 2017; and articles written in Portuguese, English or Spanish. Although the P300 has been studied over the last 50 years, in this study the analysis was focused on the recent and relevant output regarding its use as a tool for cognitive processing speed and attention assessment, since numerous good reviews have been published on early P300 studies in general.

The exclusion criteria were articles that used types of event-related potentials other than the classic P300, studies that did not include a cognitively healthy elderly group and those not having a focus on cognitive processing speed and attention.

### Search strategy

We selected studies through electronic searches on five health databases: LILACS; PsychINFO; PubMed; SCOPUS and Web of Science, using the following combination of descriptors and filters: ("P300" *OR* "electrophysiological" *OR* "evoked cognitive potentials" *OR* "evoked potential" *OR* "auditory evoked potentials") *AND* "elderly"; Activated filters: Research in humans, Aged: 65+ years, 80 and over: 80+ years.

### Study selection

Two independent "blind" reviewers conducted the search for titles and abstracts in July 2016. The studies that addressed the guiding questions were selected for full reading using a selection protocol previously created in Microsoft Excel^®^2010. In addition, a strategy of cross-reference searching was performed, by which all the references from the selected studies that addressed the guiding questions and met the inclusion criteria were selected for full reading. Reading and discussion of the studies were carried out intensively in a consensus meeting held on February 05, 2017 with the presence of authors.

**Figure 1:**
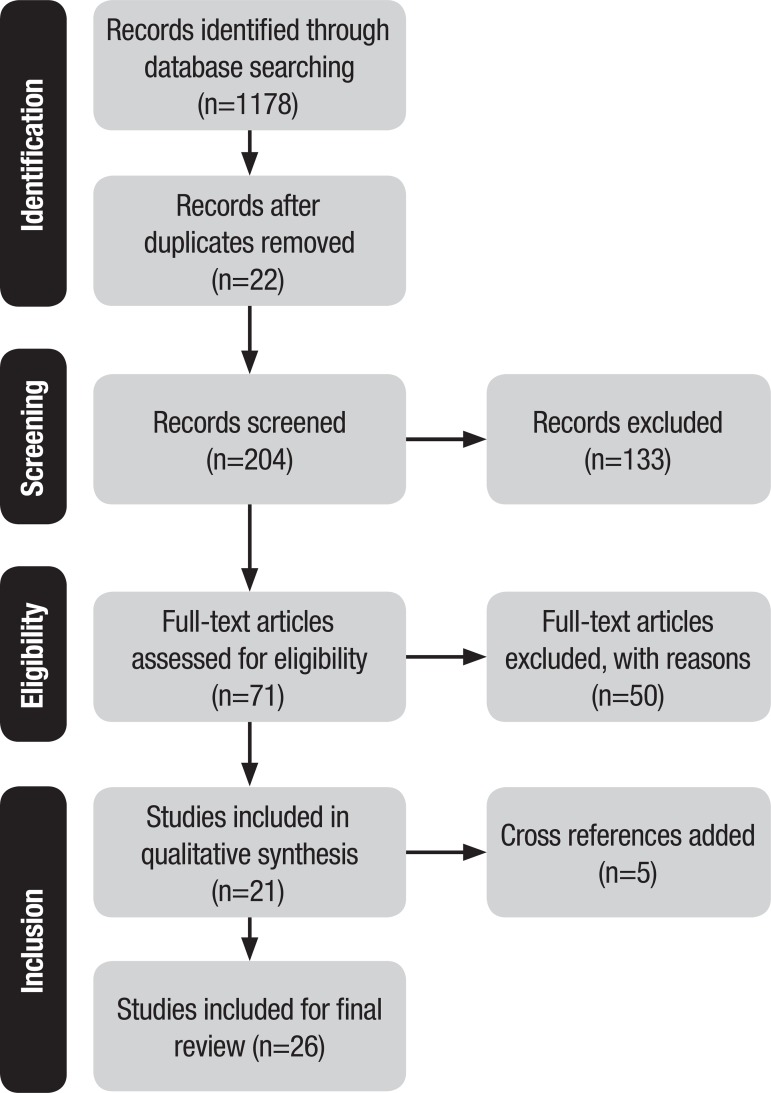
Summary of the study search and selection procedure. São Carlos, 2017.

Figure 1 depicts a summary of the steps for the study identification and selection procedure with numerical data.

### Data collection, analysis and synthesis of results

For the analysis and extraction of the data from the articles retrieved, a form devised by the authors collecting the following information was used: authors; publication year; country; study design; aims; sociodemographic information on the sample; auditory or visual ERP; complementary evaluation; statistical tests used; and main findings. The information was gathered in spreadsheets. The quantitative data were presented as simple frequency, percentage and mean. The qualitative analyses of the studies were carried out together with all the authors and presented in text and tables.

As a secondary and complementary result, the studies that reported amplitude and latency information for the healthy elderly were described and summarized in the review.

## RESULTS

### General characteristics of the studies

Of the 26 articles selected, 22 were cross-sectional studies and four randomized clinical trials. Regarding the publication date, most were published in 2014 (30.8%) and 2013 (30.8%). The studies were conducted in 12 different countries, 26.9% in the United States, and 11.6% in Japan, Italy, Brazil, Canada, and China.

Concerning the sample characteristics, the studies included information from 1,463 participants, of which 940 were healthy elderly. Regarding participant age, 23 studies cited the mean age of the healthy elderly groups, where the lowest average found was 60.0 years^13^ and the highest was 75.8 years.^14^ The majority (n = 10) had a mean age of between 70 and 74 years.^2^^,^ 15 ^-^ 20 Two studies did not cite the mean age, only the age range of the participants, which was 15-85 years^21^ for one study and 42-78 years for the other.^22^

With regard to the type of stimulus used for obtaining the P300, 50% used auditory stimulation and 50% visual stimulation, and each study used different settings for recording the ERP. Most studies (92.3%) used complementary evaluations and 23% also included electrophysiological evaluation of waves other than the P300.

### P300 evaluation

The evaluation of P300 was used in 53.8% of the articles to identify the influence of age on cognitive processing performance.^5^^,^ 15 ^-^ 17 ^,^ 19 ^,^ 21 ^,^ 23 ^-^ 29 In 19.2% of the studies, the P300 was used to compare the electrophysiological evaluation of healthy and cognitively impaired participants,^6^^,^ 13 ^,^ 14 ^,^ 30 ^,^ 31 15.4% used electrophysiological evaluation to monitor the effects of therapies in clinical trials,^32^^-^ 35 one study evaluated the P300 according to age, depression, and cognition,^2^ one evaluated depression in post-stroke patients^22^ and one paper studied the difference between older monolingual and bilingual adults.^18^ A summary of the results is given in Table 1.

**Table 1:** Details of the 26 articles regarding authors, year, country, sociodemographic characteristics, P300 evaluation, complementary evaluation and use of P300. São Carlos, 2017.

Reference	Study design		Sample (n; age; % female)	P300 evaluation	Complementary Evaluation	Objectives
	Randomized controlled trial	Transversal				
Alperin et al. (2013) USA	X		• Elderly - high executive capacity (17; 74.6 years; 68.8%) • Young - high executive capacity (13; 22.5; 61.5%) • Young - average executive capacity (13; 22.6 years; 46.1%)	Visual oddball paradigm 128 electrodes	Executive Capacity testsAmerican National Adult Reading Test and MMSE	To analyze whether age-related differences in the task of processing irrelevant stimuli are uniform between the stages of information processing.
Alperin et al. (2014a) USA	X		• Elderly (29; 72.8 years; 51.7%) • Young (25; 22.6 years; 52%)	Visual oddball paradigm 128 electrodes	Executive Capacity testsAmerican National Adult Reading Test and MMSE	To compare the performance of P3 between young and elderly.
Alperin et al. (2014b) USA	X		• Elderly - high executive capacity (15; 73.9 years; 60.0%) • Elderly - average executive capacity (14; 71.6 years; 42.8%) • Young - high executive capacity (13; 22.5; 61.5%) • Young - average executive capacity (13; 22.6 years; 46.1%)	Visual oddball paradigm 128 electrodes	Executive Capacity testsAmerican National Adult Reading Test and MMSE	To understand the mechanisms involved in the increase of P3b in the elderly and young.
Asaumi et al. (2014) Japan	X		• Healthy elderly (12; 71.0 years; 75%) • Alzheimer's Disease (12; 74.1 years; 41.7%) • High risk (12; 74.3 years; 75%) • Low risk (12; 71.6 years; 50%)	Visual oddball paradigm 4 electrodes (Fz, Cz, Pz, Oz)	HDS-R, MMSE, CDR	To compare the healthy elderly group with AD and those for dementia risk.
Bashore et al. (2015) USA	X		• Elderly (34; 70.0 years; 0%) • Young (34; 27.0 years; 0%)	Visual oddball paradigm 4 electrodes (Fz, Cz Pz, Oz)	WAIS	To compare elderly and young processing information using the measures of P300 latency and reaction time.
Bender et al. (2014) Germany	X		• Healthy elderly (17; 72.3 years; 64.7%) • Alzheimer's Disease (19; 75.2 years; 35.3%) • Young healthy controls (17; 25.9 years; 41.2%)	Auditory oddball paradigm 32 electrodesP300 only in Pz	CERAD battery, MMSE, delayed verbal memory test, Wechsler Memory Scale, and GDS	To evaluate auditory processing impairment in the elderly with AD.
Kousaie, Philips (2017) Canada	X		• Monolingual speakers (21; 71.7 years; 85.7%) • Bilingual speakers (22; 68.7 years; 68.1%)	Visual oddball paradigm 64 electrodes + 8	MoCA	To examine whether monolingual and bilingual elderly differ in terms of behavioral performance and/or brain responses during the performance of multiple tasks.
Kuba et al. (2012) Czech Republic	X		• Whole group (150; 15 - 85 years; 54%)	Visual oddball paradigm 8 electrodes	--------------------	To evaluate the effects of aging on visual evoked potentials.
Lee et al. (2013) Korea	X		• Healthy elderly (31; 75.8 years; NR) • Alzheimer's Disease (31; 76.4 years; NR)	Auditory oddball paradigm 62 electrodes	K-BNT, MMSE, Word list memory, Constructional praxis, Word list recall, TMT-A/B, SBT-K	To evaluate the clinical implication of the P300 in AD patients.
Li et al.(2013) USA	X		• Elderly (13; 63.1 years; 46.1%) • Young (13; 23.9 years; 46.1%)	Visual oddball paradigm 64 electrodes + 6 (ear lobes and electrooculogram)	------------------	To examine the effects of age on the detection of target stimuli during the visual task and simultaneously to compare the contribution of the frontal and parietal regions in the task.
Lopes et al.(2014) Brazil	X		• Control (33; 60 years; 84.4%) • Parkinson disease (44; 64 years; 45.5%)	Auditory oddball paradigm 3 electrodes	MMSE, audiometry	To investigate the presence of P300 latency prolongation in PD and its association with the clinical stage of the disease.
Lucci et al. (2013) Italy	X		• Elderly (13; 69.9 years; 61.5 %) • Middle-aged (13; 50 years; 30.8%) • Young (13; 22.8 years; 30.8%)	Visual oddball paradigm 64 electrodes	------------------	To identify the effects of aging on inhibition processes.
Miranda et al. (2012) Brazil	X		• Elderly (60; 71.1 years; 66.7 %)	Auditory oddball paradigm 4 electrodes	MMSE, ADAs-Cog, GDS	To verify the association with age, cognition and depressive symptoms.
Nowak et al. (2016) Poland	X		• Elderly (20; 65.2 years; NR) • Young (20; 24.5 years; NR)	Auditory oddball paradigm 31 electrodes	MMSE, P1-N1, MMN	To determine the changes in electrophysiological response related to age.
O'Connell et al. (2012) Ireland	X		• Elderly (14; 70.6 years; 50%) • Young (15; 22.0 years; 33.3%)	Visual oddball paradigm 32 electrodes	HADS, MMSE	To evaluate the components P3a and P3b using EEG and fMRI simultaneously
Raggi et al. (2013) Italy	X		• Elderly (13; 63.9 years; 53.8%) • Young (14; 33.6 years; 57.1%)	Auditory oddball paradigm 5 electrodes	EHI, HDRSMMSE, Raven'sAdvanced Progressive Matrices	To identify the stability of their parameters in the elderly compared to the young.
Richardson, Bucks, and Hogan (2011) UK	X		• Elderly (14; 69.1 years; NR) • Young (13; 20.3 years; NR)	Auditory oddball paradigm 24 electrodes + ground + linked-mastoid reference	MMSE, MoCA, NART, HADS, RSPM	To examine the relationship between P3 caused by repeated new stimuli and a neuropsychological measure of intellectual function.
Saliasi et al. (2013) USA	X		• Elderly (40; 65.8 years; 50.0%) • Young (40; 19.9 years; 50%)	Visual oddball paradigm 64 electrodes	MMSE, HADS	To determine whether changes in brain activity related to age are associated with working memory.
Smart et al. (2014) Canada	X		• Elderly (23; 69.6 years; 76.9%) • Subjective cognitive decline (17; 69.4 years; 70.5%)	Visual oddball paradigm 32 electrodes	IQCODE, GDS, MATS, WAIS-IV, MIA, MAC-Q, BFI e +20	To evaluate people with objective cognitive complaints.
Speer and Soldan (2015) USA	X		• Elderly (19; 70.2 years; 89.5%) • Young (25, 20.1 years; 60%)	Visual oddball paradigm 32 scalp sites	NART, WAIS-R	To verify the existence of electrophysiological changes related to the cognitive reserve in healthy participants.
Tsolaki et al. (2015) Greece	X		• Elderly (18; 67.0 years; 52.9%) • Young (27; 33.0 years; 51.8%)	Auditory oddball paradigm 256 electrodes	MMSE, TMT, MMN, N400	To analyze differences in amplitude and latency for age and sex.
Zhang et al. (2013) China	X		• Health Elderly (40; 42-78 years; NR) • Post-stroke with depression (28; 43-76 years; NR) • Post-stroke without depression (39; 43-79 years; NR)	Auditory oddball paradigm 1 electrode (Cz)	N1 and N2 waves, serum ApoE, ApoE mRNA expression, HAMD	To analyze the influence of ApoE on post-stroke depression risk and to define markers for diagnosis.
Peth-Nui (2012) Thailand	X		• Placebo (20; 64.2 years; 75.0%) • 300 mg dose (20; 61.8 years; 65.0%) • 600 mg dose (20; 61.7 years; 45.0%)	Auditory oddball paradigm 1 electrode (Cz)	Computerized battery tests, words, and pictures tests	To determine the effect of *B. monnieri* on attention, cognitive processing, working memory, cholinergic and monoaminergic functions.
Tokuda et al. (2014) Japan	X		• Elderly (20, 62.7 year; 100%)	Auditory oddball paradigm 1 electrode (Pz)	-------------	To detect differences in cognition with ARA acid supplementation in elderly men.
Zhang et al. (2014) China	X		• Swimming (29; 64.1 years; 51.7 %) • Running (27; 65.0 years; 21.8%) • Dancing (30; 65.2 years); 53.3% • Tai Chi (28; 65.5 years; 53.5%) • Control Group (30; 64.1 years; 53.3%)	Auditory oddball paradigm 1 electrode (Cz)	SECF Cognitive Scale, HAMA, HAMD	To analyze the effects of the practice of different sports on the cognitive function of the elderly.
Wang et al. (2013) Japan	X		• Elderly (8; 62.7 years; 50%)	Auditory oddball paradigm 5 electrodes	-------------	To detect cognitive changes after Tai Chi sessions in healthy elderly.

USA: United States of America; MMSE: Mini-Mental State Examination; HDS-R: Revised Hasegawa Dementia Scale; CDR: Clinical Dementia Rating; CERAD: Consortium to Establish a Registry for Alzheimer's Disease; GDS: Geriatric Depression Scale; ADAS-Cog : Alzheimer's Disease Assessment Scale; HDRS: Hamilton Depression Rating Scale; HADS: Hospital Anxiety and Depression Scale; IQCODE: Informant Questionnaire of Cognitive Decline in the Elderly; DRS-2: Dementia Rating Scale-2nd ed; MATS: Memory & Aging Telephone Screen; WAIS: Wechsler Adult Intelligence Scale; MIA: Metamemory in Adulthood questionnaire; MAC-Q: Memory Complaints Questionnaire; BFI: Big Five Inventory; NART: National Adult Reading Task; HAMA: Hamilton Anxiety Scale; HAMD : Hamilton Depression Scale; AD: Alzheimer's disease; PD: Parkinson's disease; EEG: Electroencephalograph; fMRI: Functional magnetic resonance imaging; ARA: Arachidonic acid; ApoE: apolipoprotein E;

### P300 values

Of the 26 studies, eight reported means and standard deviations of the P300 values (amplitude and latency), as summarized in Table 2. These values are only for the healthy elderly groups, serving as a reference for discussion of the subsequent studies.

**Table 2:** P300 values in healthy elderly. São Carlos, 2017.

Study	Mean ± standard deviation	
	Amplitude (µV)	Latency (ms)
Lee et al. (2013)	6.5 ± 5.5* 4.0 ± 2.8** 4.1 ± 5.5***	362.5 ± 44.2* 362.2 ± 43.5** 385.1 ± 38.5***
Raggi et al. (2013)^a^	5.3 ± 2.6* 6.6 ± 2.7** 5.8 ± 2.3***	357.8 ± 30.9* 357.2 ± 31.3** 361.0 ± 33.7***
Smart et al. (2014)^b^	2.2 ± 0.2* 2.3 ± 0.2** 3.0 ± 0.2***	484 ± 21* 479 ± 16** 464 ± 16****
Tokuda et al. (2014)^e,g^	7.34^c^ *** 10.88^d^ ***	406^c^ *** 377^d^ ***
Tsolaki et al. (2015)^g^	3.5***	428***
Wang et al. (2013)^e^	18.5 ± 9.2* 15.2 ± 4.4** 17.9 ± 3.3***	351.9 ± 32.2* 348.8 ± 33.9** 355.9 ± 31.3***
Zhang, Ni and Chen (2014)^e, f^	5.2 ± 1.9**	340.4 ± 23.7**
Zhang et al. (2013)	8.6 ± 2.9**	320.0 ± 20.2**

a Values related to the ISI 800ms evaluation;

b Values related to the Go stimulus;

c Values related to healthy elderly with low arachidonic acid concentration;

d Values related to healthy elderly with high arachidonic acid concentration;

e Values related to baseline;

f Control group;

g studies did not report standard deviation from the mean.

* Fz electrode;

** Cz electrode;

*** Pz electrode;

**** FCz electrode.

The statistical procedures most used for the treatment of P300 data were those recommended when the data adhere to normality. The variance analysis by ANOVA was the most used (61.5%), the *t*-test was the most used procedure for comparison of groups and therapy effects (28%), and the non-parametric option predominantly used to compare the ranking between groups was the Mann-Whitney test. The Spearman (16%) and Pearson (8%) correlation coefficients were used for correlation analyses.

Regarding the most evident findings, half of the articles found an influence of age on P300 values. In 19.2% of the studies, associations between neurocognitive impairment and worse electrophysiological evaluation^6^^,^ 13 ^,^ 14 ^,^ 30 ^,^ 31 were found. Education was an item commonly not associated with P300 evaluation.^2^^,^ 16 ^,^ 27 Two articles presented contrasting results for the association between sex and P300 values - the first study found that latency in the male group was greater with advancing years^21^ whereas the second found that response to the stimulus in the frontal region was faster in men.^5^ Four studies performed an association analysis between neuropsychological and cognitive tests with P300 values, although two of these failed to identify a relationship between the two variables. In both studies that demonstrated this association, the authors confirmed a relationship between P300 and the working memory domain in healthy elderly^29^ and for general scores on word list recognition, constructional praxis, and word fluency neuropsychological tests of the MMSE and CERAD-K in elderly with Alzheimer's disease.^14^ Finally, one study examined the difference between monolingual and bilingual elderly in terms of brain response during the performance of multiple tasks.^18^

The amplitude and latency values of P300 in healthy elderly are given in Table 2, showing no pattern of response to the stimuli among the studies described. The amplitude values ranged from 2.2 µV (±0.2)^31^ to 18.5 µV (±9.2)^34^ and latency values from 320 ms (±20.2)^22^ to 484 ms (±21),^31^ independently of the channel (electrode) used to capture the response to the stimulus.

## DISCUSSION

### P300: sociodemographics and values in healthy elderly

Of the cross-sectional studies that included a control group, 14 evaluated the effects of healthy aging on endogenous evoked potentials.^5^^,^ 15 ^-^ 17 ^,^ 19 ^-^ 21 ^,^ 23 ^-^ 29 In all studies, age influenced cognitive processing. These data corroborate a review study that indicated a decrease in P300 amplitude at advanced ages.^9^ Another integrative review reported that latency tends to increase, and amplitude to decrease, with advancing age.^36^

On average, the P300 latency increases by around 2 ms per year, as wave amplitude is linearly reduced in older people.^21^ The data found corroborates the findings of a study that estimated the latency effect of P300 in 62 healthy elderly subjects and subdivided them into three groups according to age: 60-64 years, 65-69 years and 70-74 years. The researchers found a linear significant increase of 2.85 ms per year in the age groups 65-69 years and 70-74 years.^8^

The articles also showed that the P300 amplitude values ranged from 2.2 µV to 18.5 µV and the latency values from 320 ms to 484 ms. These variations found may be related to the characteristics of the samples studied, the variables analyzed and the methods adopted in each study.

The increase in wave amplitude may result in increased use of frontal executive functions as a compensatory mechanism to adequately perform the tasks.^16^ The reaction time to the stimuli differed between elderly and adults groups. For the elderly, it was a mean of 555 ms, in middle-age adults 515 ms while the average among the young was 480 ms.^25^ In one of the studies, poor performance on tasks involving working memory was associated with a higher latency value in elderly, and in relation to the amplitude, compared to the young, the elderly had higher values in frontal regions and smaller values in posterior regions.^29^ In the elderly, the activity is more distributed in the temporal and superior temporal lobes.^5^ Besides the changes related to age, differences were identified in the parietal and frontal regions.^24^^,^ 28

One of the articles analyzed showed that the changes in P300 are more pronounced in men, where latency increased by 2.3 ms/year in men and 1.6 ms/year in women.^21^ However, on topographic imaging exams, elderly men had greater distribution in the frontal region compared to women, responding to the stimulus faster.^7^

Education and cognitive tests showed no relationship with the results of the electrophysiological evaluations. However, the young had better performance on the MMSE^17^ and had over 10 years more education than the elderly,^27^ a factor which may have influenced age-related P300 results. These findings corroborate the results of the study by Miranda et al. (2012)^2^ in which no correlation was found between increase in P300 latency and education in elderly with hearing loss. A study carried out in Colombia of healthy elderly revealed an inverse relationship between latency and amplitude values; and, regarding gender and education, no differences were observed for the latency and amplitude of the P300 wave.^37^ One study found that bilingual elderly performed better than monolingual elderly on the electrophysiological measures during cognitive tasks.^18^

### P300: comparison between healthy elderly and those with mental disorders

Regarding the use of P300 in mental disorders, most of the studies had used event-related potentials to evaluate whether differences existed between cognitively healthy elderly and subjects with cognitive loss and/or Alzheimer's disease (AD).

Three studies found that patients with AD had lower amplitude of the P300 wave when compared to healthy elderly.^6^^,^ 14 ^,^ 30 Although latency did not differ between the two groups in the study by Lee et al.,^14^ it was higher for elderly with AD compared to healthy elderly with high and low risk of dementia in the study of Asaumi et al. (2014).^6^ In addition to these investigations, another study observed better latency and amplitude values for the group without subjective memory complaints than the group with complaints.^31^ In contrast, another investigation,^2^ despite identifying greater latency with increasing age, found no correlation of the P300 results with cognitive evaluation tests and depressive symptoms in elderly with hearing loss.

The relationship of P300 with cognitive loss and AD has been previously described in other studies. Since the P300 response is related to fundamental elements of cognitive function, it may be useful in the early diagnosis of dementia, serving as a complementary tool to existing instruments.^11^

A literature review evaluated whether the P300 was able to estimate the risk of progression from mild cognitive impairment (MCI) to AD. Eight studies were evaluated and found that the electrode positioned in the parietal region is the most effective to evaluate this progression, evidencing increased latency and decreased amplitude. However, despite being a promising method for evaluating this progression, such investigations remain scarce, with small samples and heterogeneous results. This demonstrates a need for further studies so that this tool can be used in clinical practice.^38^ Another meta-analysis of 13 papers investigated whether the P300 can serve as a useful neurophysiological marker to discriminate MCI and predict its progression. Differences were identified in P300 latency of patients with MCI when compared to controls and to patients with AD, indicating that the this may be useful for identifying early cognitive impairment and its progression, including for AD.^4^ A meta-analysis of twenty studies sought to characterize the P300 in probable AD compared with healthy controls and found that the amplitude was lower in individuals with AD.^39^ Another systematic review of eight investigations found a consensus that the P300 latency is increased in elderly with AD, but no consensus on amplitude (which may also be due to the methodological variations of the studies assessed).^36^

One of the evaluated articles in this review analyzed the association of P300 latency with the clinical stage of Parkinson's disease (PD). Latency values of the elderly with PD that exceeded two units from the standard deviation of the latency values of the healthy subjects were considered altered. In the group at initial stages of PD, 10% were considered altered and 31% at the advanced stages, showing an association between PD severity and increased P300 latency.^13^ The fact that the P300 serves as a possible predictor of PD evolution based on higher latency has been described previously.^40^

Another mental disorder found in the present review was depression. Zhang et al. (2013)^22^ evaluated stroke patients with and without depression versus a control group. The P300 latency values were higher, and amplitude values lower, in post-stroke patients with depression compared to the no depression and healthy groups. These data corroborate a study conducted in Australia that found greater amplitude and lower latency in patients with depression compared to those without depression,^41^ and also another in India, that found a delay in latency among patients with depression compared to healthy controls, which was proportional to disease severity.^42^

### P300 in clinical trials with healthy elderly

Four trials with a total of 232 participants^32^^-^ 35 used the P300 measurement to identify the effects of the use of Bacopa monnieri^32^ and arachidonic acid,^33^ Tai Chi practice^34^ and several sports modalities^35^ on cognitive processing in healthy elderly compared to controls. In all studies, the evaluation of P300 was sensitive for detecting the effects of therapies. Protocols ranged from 12 weeks to 18 months. These trials yielded important information, such as: when the treatment was interrupted, the P300 values returned to similar values to those before treatment began.^32^ During the therapy period, the control group of elderly continued to show increased latency and decreased amplitude compared to the elderly undergoing intervention,^35^ the electrophysiological evaluation may be improved with the use of other waves, such as Mismatch Negativity (MMN).^34^

One of the trials indicated that Pz would be a more sensitive electrode for detecting the effects of cognitive processing interventions.^34^ One of the articles reported information only for the electrode Pz^33^ while another only for the electrode Cz.^35^ Perhaps the sensitivity of the electrodes for detecting the effects depends on the characteristics of the sample of participants and type of intervention used. Therefore, it is up to the authors to decide what information from which electrode should be analyzed. Future studies could focus on the sensitivity of each electrode.

In the literature, other studies involving participants with different characteristics used the P300 to detect effects on cognitive processing of drug therapies, such as levetiracetam in the treatment of epilepsy,^43^ use of perospirone in people with schizophrenia^44^ and donepezil in the elderly with vascular dementia type.^45^ The P300 has also proved sensitive in clinical trials involving additional and complementary therapies, such as vitamin B12 supplementation^46^ and acupuncture.^47^

In clinical trials for the diagnosis of disorders, the P300 appears to be useful for identifying cognitive loss, age-related or otherwise, cognitive changes after complex surgical procedures and diagnoses for Alzheimer's disease.^36^ In other types of controlled clinical trials, the P300 was effective in identifying the influence of unhealthy habits, such as smoking.^48^ In summary, the electrophysiological evaluation by P300 seems to be a useful and sensitive tool to detect effects on cognitive processing in therapies with clinical trials and in studies of accuracy for diagnosing disorders.

### Main findings of the study

This review aimed to systematize the latest information related to the use of P300 as a tool for cognitive processing assessment in healthy elderly. We found that:

- Most of the cross-sectional studies sought to identify the influence of age on endogenous ERP and showed an increase in latency and a decrease in amplitude with increasing age, due to the natural aging process. In elderly with cognitive loss, Alzheimer's disease, Parkinson's disease and depression, the increase in latency is more pronounced, including progressive decline according to the degree of cognitive impairment. There was no consensus on the reduction in amplitude in these cases.
- No relationship between the P300 and education was observed. Moreover, bilingual elderly performed better than monolingual elderly. However, there were no subjects with low education in the articles analyzed.
- The association between gender and P300 values is not well described in the literature. Some results indicate that men have greater latency with advancing years and faster response to frontal stimulus compared to women.
- This review was unable to indicate the normative values of amplitude and latency in healthy elderly. Among the eight studies that reported this information, the values ranged from 2.2 µV to 18.5 µV for amplitude, and from 320 ms to 484 ms for latency.
- Regarding visual and auditory evaluation, there are marked differences in the number of channels, electrode locations, impedance values, the number of rare and frequent stimuli used, stimulation differences regarding images (color, distance, type, size) or sounds (frequency, duration), among others.
- The focus of this review was healthy elderly. It was observed in all the articles that this group was used as a control for another group: young; elderly with mild cognitive impairment, Alzheimer's disease, Parkinson's disease, depression; or elderly receiving some intervention. Therefore, there is a lack of studies that specifically evaluated healthy elderly as the main focus.

Based on these results, further investigations should be conducted in order to:

- Evaluate the relationship between education and the P300 in elderly with different levels of literacy.
- Clarify the relationship of gender and education with variations in evoked potentials.
- Standardize the parameters used to evaluate and capture endogenous evoked potentials, allowing greater comparison between studies.
- Include healthy elderly as the main investigation group (and not only as a control group), following the evolution of this group over the years through longitudinal studies.

In conclusion, the reviewed scientific evidence suggests that there is an increase in latency and a decrease in amplitude with advancing age. Latency impairment is more pronounced in individuals with mental disorders, but no consensus exists with respect to amplitude. Normative values of amplitude and latency in healthy elderly cannot be established due to the variability in methodological characteristics, such as number of channels, electrode locations, impedance values, and differences in stimulation procedures. Thus, there is no evidence on the association between gender, education and the P300 in healthy elderly. In clinical trials, the P300 has proven a useful tool to evaluate the effects of interventions in the elderly, but most studies used healthy elderly only as a control group.

Based on these results, it is suggested that further investigations should be conducted to evaluate the true relationship between age, education, gender and the P300 in elderly. Finally, as this review identified a lack of studies designed to specifically investigate cognitive processing in healthy aging using the P300, it is recommended that future studies include healthy elderly as the main investigation group.
